# Efficacy and safety of controlled-release oxycodone/naloxone versus controlled-release oxycodone in Korean patients with cancer-related pain: a randomized controlled trial

**DOI:** 10.1186/s40880-017-0241-4

**Published:** 2017-09-11

**Authors:** Kyung-Hee Lee, Tae Won Kim, Jung-Hun Kang, Jin-Soo Kim, Jin-Seok Ahn, Sun-Young Kim, Hwan-Jung Yun, Young-Jun Eum, Sung Ae Koh, Min Kyoung Kim, Yong Sang Hong, Jeong Eun Kim, Gyeong-Won Lee

**Affiliations:** 1 Department of Hematology-Oncology, Yeungnam University Medical Center, Daegu, 42415 South Korea; 2 Department of Oncology, Asan Medical Center, University of Ulsan, Seoul, 05505 South Korea; 3 Department of Hematology-Oncology, Gyeongsang National University Hospital, Jinju, 52727 South Korea; 4 Department of Internal Medicine, SMG-SNU Boramae Medical Center, Seoul, 07061 South Korea; 5 Division of Hematology-Oncology, Department of Medicine, Samsung Medical Center, Seoul, 06351 South Korea; 6 Department of Hematology-Oncology, National Cancer Center, Ilsan, 10408 South Korea; 7 Department of Hematology-Oncology, Chungnam National University Hospital, Daejeon, 35015 South Korea; 8 Medical Affairs, Mundipharma Korea Ltd, Seoul, 04637 South Korea; 9 Department of Oncology, Asan Medical Center, University of Ulsan College of Medicine, 388-1, Pungnap-dong, Songpa-gu, Seoul, 138-736 South Korea

**Keywords:** Constipation, Naloxone, Oxycodone, Quality of life, Safety

## Abstract

**Background:**

Controlled-release oxycodone/naloxone (OXN-CR) maintains the effect of opioid-induced analgesia through oxycodone while reducing the occurrence rate of opioid-induced constipation through naloxone. The present study was designed to assess the non-inferiority of OXN-CR to controlled-release oxycodone (OX-CR) for the control of cancer-related pain in Korean patients.

**Methods:**

In this randomized, open-labeled, parallel-group, phase IV study, we enrolled patients aged 20 years or older with moderate to severe cancer-related pain [numeric rating scale (NRS) pain score ≥4] from seven Korean oncology/hematology centers. Patients in the intention-to-treat (ITT) population were randomized (1:1) to OXN-CR or OX-CR groups. OXN-CR was administered starting at 20 mg/10 mg per day and up-titrated to a maximum of 80 mg/40 mg per day for 4 weeks, and OX-CR was administered starting at 20 mg/day and up-titrated to a maximum of 80 mg/day for 4 weeks. The primary efficacy endpoint was the change in NRS pain score from baseline to week 4, with non-inferiority margin of −1.5. Secondary endpoints included analgesic rescue medication intake, patient-reported change in bowel habits, laxative intake, quality of life (QoL), and safety assessments.

**Results:**

Of the ITT population comprising 128 patients, 7 with missing primary efficacy data and 4 who violated the eligibility criteria were excluded from the efficacy analysis. At week 4, the mean change in NRS pain scores was not significantly different between the OXN-CR group (*n* = 58) and the OX-CR group (*n* = 59) (−1.586 vs. −1.559, *P* = 0.948). The lower limit of the one-sided 95% confidence interval (−0.776 to 0.830) for the difference exceeded the non-inferiority margin (*P* < 0.001). The OXN-CR and OX-CR groups did not differ significantly in terms of analgesic rescue medication intake, change in bowel habits, laxative intake, QoL, and safety assessments.

**Conclusions:**

OXN-CR was non-inferior to OX-CR in terms of pain reduction after 4 weeks of treatment and had a similar safety profile. Studies in larger populations of Korean patients with cancer-related pain are needed to further investigate the effectiveness of OXN-CR for long-term pain control and constipation alleviation.

*Trial registration* ClinicalTrials.gov NCT01313780, registered March 8, 2011

## Background

Cancer-related pain is estimated to be prevalent in 15% to more than 75% of cancer patients, depending on the type and extent of the malignancy as well as several other factors [1–3]. Cancer-related pain has a significant effect on patient quality of life (QoL) and is a clinically important indicator of tumor progression [4]. The European Society for Medical Oncology clinical practice guidelines support the use of opioids as a treatment option for patients with moderate to severe cancer-related pain; however, constipation is a common and persistent adverse effect in opioid therapy, and patients rarely develop tolerance to this condition [4].

The primary cause of opioid-induced constipation is the stimulation of δ-, κ-, and μ-opioid receptors, particularly the μ subtype, in the gastrointestinal tract, which reduces bowel tone and contractility and extends gastrointestinal transit time [5–8]. In some patients, laxatives effectively alleviate opioid-induced constipation; however, because these do not target the underlying cause, constipation remains a significant clinical problem for many patients with cancer-related pain who were treated with opioids and a barrier to achieving optimal pain control [6].

A controlled-release formulation of oxycodone (OX-CR), an opioid analgesic, is available in many countries. A different controlled-release formulation has recently been developed that combines oxycodone with naloxone. The oxycodone/naloxone controlled-release formulation (OXN-CR) was designed to reduce the occurrence rate of opioid-induced constipation through local antagonistic effects of naloxone on peripheral μ-opioid receptors in the gastrointestinal tract [9]. By competitively binding to μ-opioid receptors on neurons within the myenteric plexus of the gastrointestinal tract, naloxone prevents oxycodone from exerting its effect on the gastrointestinal system, thereby minimizing the risk of constipation [7, 10]. Furthermore, because of its low oral bioavailability (<3%), naloxone has a minimal effect on opioid receptors in the central nervous system, thereby sparing the centrally mediated analgesic efficacy of oxycodone [5, 9, 11].

In a previous 4-week, international, multicenter, double-blinded, randomized trial with a predominantly (>99%) Caucasian population of patients with cancer-related pain, OXN-CR significantly improved bowel function compared with OX-CR (*P* < 0.01) and reduced the mean total laxative intake by 20% while providing a similar level of analgesic efficacy [12]. Inter-individual variability in opioid-induced constipation has been described [13, 14], suggesting the need for studies on opioid-induced constipation in a range of patient populations. To date, no published studies have reported a direct comparison of OXN-CR and OX-CR in Asian patients with cancer-related pain. In the present study of Korean patients with cancer-related pain, we compared OXN-CR with OX-CR in terms of analgesic efficacy, occurrence rate of constipation, and safety.

## Patients and methods

### Study design and patients

This 4-week, multicenter, open-labeled, randomized, parallel-group, active-control study (ClinicalTrials.gov: NCT01313780) was conducted between May 19, 2011 and November 18, 2013 at seven oncology/hematology centers in Korea. Inclusion criteria were as follows: patient at least 20 years old; moderate to severe cancer-related pain [i.e., a numeric rating scale (NRS) pain score ≥4] that required continuous treatment with a strong opioid analgesic; and opioid-naïve or previously received only weak opioids, or not treated with naloxone or strong opioids (except for occasional as-required use) within 4 weeks before the screening visit. Patients were excluded from the study if they met any of the following criteria: treatment with OXN-CR or OX-CR within 4 weeks or chemotherapy or radiotherapy within 2 weeks before the screening visit; predominantly non-cancer-related pain; treatment with stable doses of laxatives for 1 week or more before the screening visit; major surgery within 1 month before the screening visit or planned surgery; or clinically significant non-cancerous gastrointestinal disease or significant structural abnormalities of the gastrointestinal tract and significant cardiovascular, respiratory, renal, or hepatic impairment.

### Ethics, consent, and permissions

The present study was performed in compliance with Good Clinical Practice guidelines and in accordance with the principles set forth in the Declaration of Helsinki. The study protocol was approved by the institutional review board at each site. All patients provided written informed consent prior to participation in the study.

### Treatment

Patients were randomized (1:1) using computer-generated randomization lists to receive either OXN-CR tablets or OX-CR tablets, which were taken orally for 4 weeks. The starting dose of OXN-CR was 20 mg/10 mg per day and that of OX-CR was 20 mg/day. Up-titration of OXN-CR (to 80 mg/40 mg per day) and OX-CR (to 80 mg/day) was permitted at the discretion of the investigator for the following reasons: use of analgesic rescue medication at least twice daily; increased NRS pain score compared with that on the previous visit; or inadequate pain control at the existing dose.

An immediate-release formulation of oxycodone (maximum 10 mg/day) was provided as the analgesic rescue medication. Magnesium oxide (MgO) was prescribed by the study investigators as the laxative rescue medication. Patients were instructed on when to take the laxative and to discontinue its use once constipation symptoms resolved.

Patients were permitted to continue analgesics (non-steroidal anti-inflammatory drugs, weak opioid analgesics, and adjuvant analgesics) that were being used at a stable dose prior to the screening visit. The use of opioid antagonists (e.g., single-ingredient naloxone or naltrexone), stimulant laxatives, enemas, lubricants, and other medications affecting gastrointestinal movement were prohibited during the study.

### Study assessments

At baseline, week 1, and week 4, patients were asked to indicate the average severity of their pain over the previous 24 h, according to the 11-point NRS, ranging from 0 (“no pain”) to 10 (“unbearable/severe pain”) [15]. The primary efficacy endpoint was the change in NRS pain score from baseline to week 4.

Secondary endpoints included dose, duration of use, and administration frequency of analgesic rescue medication (immediate-release oxycodone) and laxative rescue medication (MgO) used during the study; change in bowel habits from baseline to week 4 as rated by patients according to a three-point Likert scale (“worsened,” “no change”, or “improved”) [16]; and change in QoL from baseline to week 4, as assessed using the self-administered European Organization for the Research and Treatment of Cancer Quality of Life Questionnaire-C30 (EORTC QLQ-C30) [17]. Adherence to treatment was assessed at each visit by comparing the dose of unused study drugs returned by the patient against the prescribed dose. The adherence rate was computed as follows: adherence rate = total number of doses actually administered/total number of doses prescribed × 100%.

Adverse events (AEs), defined as any undesirable and unintended sign, symptom, or disease temporally associated with the use of OXN-CR and OX-CR, which may or may not be related to both drugs, were reported at each study visit after randomization and graded using the Common Terminology Criteria for Adverse Events v4.0 [18]. An adverse drug reaction (ADR) was defined as an AE that was possibly related to OXN-CR or OX-CR. Clinically significant abnormalities in clinical laboratory tests, electrocardiogram (ECG) data, and vital signs were also assessed.

### Statistical analysis

An estimated sample size of 51 patients per treatment group was required to provide a 81% power to detect non-inferiority using a one-sided, two-sample *t* test with a margin of equivalence of −1.5 and an α of 0.05. Assuming a dropout rate of 20%, it was estimated that 64 patients per treatment group would need to be randomized.

Efficacy endpoints were analyzed for the full analysis set (FAS) population (i.e., all patients with at least one measurement of primary efficacy after treatment, excluding patients not meeting the eligibility criteria). AE data were analyzed for the safety analysis population (i.e., all patients who received at least one dose of OXN-CR or OX-CR). Demographic data and laboratory measurements were analyzed for the FAS population. All analyses were conducted using available data, and no imputation was performed for missing data except for the analysis of the dose for OXN-CR or OX-CR where missing data were handled using last-observation-carried-forward analysis.

The one-sided 95% confidence interval (CI) for the difference in change in NRS pain score from baseline to week 4 between the OXN-CR and OX-CR groups was calculated to determine the non-inferiority of OXN-CR with respect to OX-CR. If the lower limit of the one-sided 95% CI for the difference between OXN-CR and OX-CR groups was −1.5 or higher, OXN-CR was considered non-inferior to OX-CR.

A *t* test, analysis of variance (ANOVA), or the Mann–Whitney *U* test was used for continuous variables. The Chi square test, Fisher’s exact test, or Mantel–Haenszel test, using a stratification factor when appropriate, was used for categorical variables. Repeated-measures ANOVA was used to analyze data at different time points and to compare the OXN-CR and OX-CR groups. The percentage of patients experiencing at least one AE was calculated for each treatment group. All statistical analyses were performed using a two-sided test with a significance level of 0.05, unless otherwise stated. All analyses were performed using SAS software (SAS Institute Inc., Cary, NC, USA).

## Results

### Patient disposition and characteristics

**Fig. 1:**
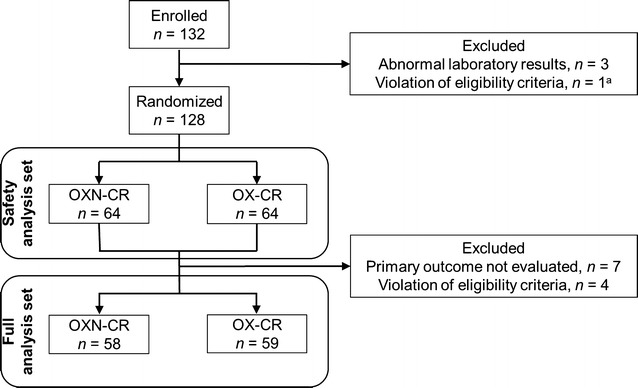
Flowchart of patients with cancer-related pain who were randomized to receive either controlled-release oxycodone/naloxone (OXN-CR) or controlled-release oxycodone (OX-CR). ^a^The patient was found to have significant structural/functional abnormalities of the gastrointestinal tract which was deemed to be not appropriate for oral medicine administration

Patient disposition is shown in Fig. 1. One hundred thirty-two patients were enrolled and screened. Of these, three had abnormal laboratory results and one violated the eligibility criteria and were therefore excluded. The remaining 128 patients were randomized to receive OXN-CR (*n* = 64) or OX-CR (*n* = 64) and were included in the intention-to-treat (ITT) population and the safety analysis population. Seven patients with missing primary efficacy data and four patients who violated the eligibility criteria were excluded from the efficacy analysis, leaving 117 patients in the FAS population. Of these, 53 patients (25 in the OXN-CR group and 28 in the OX-CR group) discontinued from the study; the remaining 64 patients (33 in the OXN-CR group and 31 in the OX-CR group) completed the study. Patient demographics and clinical characteristics at baseline are shown in Table 1. The mean ages of patients in the OXN-CR and OX-CR groups were 60.8 ± 11.1 and 60.1 ± 10.4 years, respectively (*P* = 0.709). Both groups were generally similar in terms of demographic characteristics, clinical characteristics, and prior analgesic use at baseline.

**Table 1:** Demographics and clinical characteristics of patients with cancer-related pain who were treated with controlled-release oxycodone/naloxone (OXN-CR) or controlled-release oxycodone (OX-CR) (full analysis set, FAS)

Variable	OXN-CR	OX-CR	*P* value
Total (cases)	58	59	
Age, years [cases (%)]			0.297
≥70	17 (29.3)	11 (18.6)	
<70	41 (70.7)	48 (81.4)	
Sex [cases (%)]			0.343
Male	43 (74.1)	39 (66.1)	
Female	15 (25.9)	20 (33.9)	
Cancer type [cases (%)]^a^			NA
Colorectal	24 (41.4)	23 (39.0)	
Gastric	10 (17.2)	8 (13.6)	
Lung	6 (10.3)	5 (8.5)	
Pancreatic/hepatic bile duct	6 (10.3)	9 (15.3)	
Prostate	2 (3.4)	3 (5.1)	
Esophageal	3 (5.2)	1 (1.7)	
Others	8 (13.8)	11 (18.6)	
Metastasis [cases (%)]			0.990
Yes	57 (98.3)	58 (98.3)	
No	1 (1.7)	1 (1.7)	
Prior analgesic use (number of times of administration)^b^			NA
Strong opioids	23	22	
Weak opioids	39	39	
NSAID/acetaminophen	11	12	
Adjuvant analgesics^c^	1	0	
Other medications^d^	1	0	

*NSAID* non-steroidal anti-inflammatory drug, *NA* not applicable

^a^The percentage in either group does not add up to 100% because one patient in the OXN-CR group had colorectal cancer and sarcoma and one patient in the OX-CR group had gastric cancer and lung cancer

^b^Analgesics were administered 2–6 months prior to the screening visit

^c^“Adjuvant analgesics” (pregabalin) was administered once to one patient in the OXN-CR group

^d^“Other medications” (codeine) was administered once to one patient in the OXN-CR group

### Exposure and adherence to treatment

The mean daily doses of oxycodone administered were 29.7 ± 15.2 mg in the OXN-CR group and 26.9 ± 13.8 mg in the OX-CR group (*P* = 0.308). At week 4, adherence to treatment was slightly higher in the OXN-CR group than in the OX-CR group, but not significantly different (95.7% vs. 89.8%, *P* = 0.190).

### Pain score

**Fig. 2:**
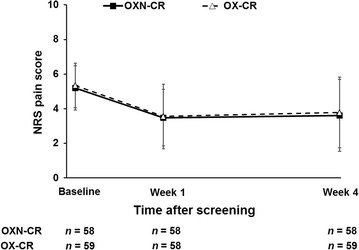
Reduction in pain scores from baseline to week 4 in the OXN-CR and OX-CR groups (full analysis set, FAS). The *error bars* represent standard deviation (SD). *NRS* numeric rating scale

Patients in both the OXN-CR and OX-CR groups had similar reductions in NRS pain scores from baseline (Fig. 2). In the FAS population, the mean changes in the NRS pain score from baseline to week 4 were not significantly different between the OXN-CR and OX-CR groups (−1.586 ± 2.217 vs. −1.559 ± 2.215, *P* = 0.948). The one-sided 95% CI for the difference between treatment groups was −0.776 to 0.830 (mean value of difference 0.027; *P* < 0.001). The lower limit of the 95% CI exceeded the non-inferiority margin of −1.5; therefore, OXN-CR was deemed non-inferior to OX-CR.

### Secondary endpoints

#### Analgesic rescue medication intake

Analgesic rescue medication (immediate-release oxycodone) was used by 86.2% (50/58) of patients in the OXN-CR group and 83.1% (49/59) of patients in the OX-CR group. Both groups did not differ significantly in terms of the administration frequency, daily dose, and duration of rescue analgesic medication intake. Immediate-release oxycodone was administered 127 times in the OXN-CR group and 145 times in the OX-CR group. The mean daily dose was 9.19 mg in the OXN-CR group and 10.19 mg in the OX-CR group (*P* = 0.535), and the corresponding mean durations of immediate-release oxycodone intake were 15.7 and 11.4 days, respectively (*P* = 0.230). The groups did not differ significantly in terms of administration frequency, daily dose, and duration of analgesic rescue medication intake.

#### Bowel habits and laxative intake

**Fig. 3:**
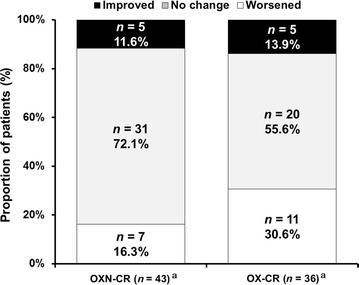
Patient-reported change in bowel habits from baseline to week 4 in OXN-CR-treated patients and OX-CR-treated patients (FAS). ^a^Patients with available data at week 4

A total of 43 patients in the OXN-CR group and 36 patients in the OX-CR group rated their bowel habit changes at week 4 and provided available data for analysis. Bowel habit changes were not significantly different between the two groups (*P* = 0.264). Most patients reported “no change” in bowel habits between baseline and week 4 [31 (72.1%) patients in the OXN-CR group vs. 20 (55.6%) patients in the OX-CR group] (Fig. 3). Fewer OXN-CR-treated patients reported “worsened” bowel habits than OX-CR-treated patients [7 (16.3%) vs. 11 (30.6%)]. Bowel habit changes were similar between the two groups when patients with a history of colorectal cancer were excluded (*P* = 0.294).

During the study, 29 (50.0%) patients in the OXN-CR group and 31 (52.5%) patients in the OX-CR group received the laxative rescue medication (MgO). No significant differences were observed in the administration duration, daily dose, and total dose of MgO between the treatment groups.

#### QoL assessments

A total of 36 patients in the OXN-CR group and 31 patients in the OX-CR group completed EORTC QLQ-C30 at week 4 and provided available data for analysis. Mean scores for all QoL measures were low at baseline and at week 4 (Table 2). Changes in scores from baseline to week 4 for all QoL measures were minimal and were similar between treatment groups.

**Table 2:** Patient-reported change in EORTC QLQ-C30 scores from baseline to week 4 in the OXN-CR and OX-CR groups (FAS)

Variable	Score at baseline		Score at week 4		Score change from baseline to week 4			
	OXN-CR (*n* = 58)	OX-CR (*n* = 59)	OXN-CR (*n* = 36)^a^	OX-CR (*n* = 31)^a^	OXN-CR (*n* = 36)^a^	*P* value	OX-CR (*n* = 31)^a^	*P* value
Global health status	3.74 ± 1.19	3.49 ± 1.34	3.88 ± 1.38	3.65 ± 1.21	−0.01 ± 1.34	0.951	−0.16 ± 1.50	0.554
Functional scales								
Physical functioning	1.89 ± 0.56	2.23 ± 0.67	2.14 ± 0.70	2.21 ± 0.67	0.29 ± 0.54	0.002	0.09 ± 0.54	0.360
Role functioning	1.85 ± 0.77	2.40 ± 1.03	2.21 ± 0.86	2.42 ± 0.99	0.44 ± 0.87	0.002	0.24 ± 0.88	0.126
Emotional functioning	1.72 ± 0.58	1.96 ± 0.65	1.86 ± 0.59	2.06 ± 0.84	0.15 ± 0.48	0.073	0.19 ± 0.69	0.241
Cognitive functioning	1.53 ± 0.55	1.85 ± 0.63	1.92 ± 0.65	1.95 ± 0.85	0.35 ± 0.77	0.011	0.13 ± 0.59	0.280
Social functioning	1.92 ± 0.70	2.12 ± 0.96	2.03 ± 0.70	2.15 ± 0.94	0.21 ± 0.85	0.200	0.11 ± 0.72	0.512
Symptoms scales/items								
Fatigue	2.13 ± 0.68	2.41 ± 0.76	2.44 ± 0.70	2.42 ± 0.80	0.35 ± 0.68	0.004	0.16 ± 0.74	0.234
Nausea and vomiting	1.49 ± 0.73	1.63 ± 0.67	1.67 ± 0.85	1.68 ± 0.80	0.21 ± 0.65	0.055	0.03 ± 0.87	0.904
Pain	2.35 ± 0.70	2.69 ± 0.71	2.44 ± 0.72	2.65 ± 0.95	0.14 ± 0.82	0.319	0.06 ± 0.85	0.831
Dyspnea	1.48 ± 0.71	1.86 ± 0.86	1.89 ± 0.89	2.16 ± 0.90	0.42 ± 0.81	0.006	0.32 ± 0.87	0.058
Insomnia	2.17 ± 0.92	2.20 ± 1.00	2.14 ± 0.87	2.19 ± 1.01	0.03 ± 1.08	0.944	0.10 ± 0.83	0.666
Appetite loss	2.05 ± 0.87	2.34 ± 1.01	2.31 ± 1.06	2.35 ± 1.05	0.25 ± 1.02	0.188	0.13 ± 0.99	0.596
Constipation	1.74 ± 0.85	1.81 ± 0.90	1.94 ± 0.83	2.26 ± 1.00	0.25 ± 0.81	0.081	0.48 ± 0.93	0.009
Diarrhea	1.26 ± 0.58	1.49 ± 0.70	1.42 ± 0.73	1.23 ± 0.43	0.11 ± 0.92	0.505	−0.23 ± 0.50	0.039
Financial difficulties	1.97 ± 0.86	2.15 ± 1.06	2.17 ± 0.97	2.16 ± 0.93	0.19 ± 1.04	0.231	−0.03 ± 0.60	1.000

Results are presented as mean ± standard deviation

*EORTC QLQ-C30* European Organization for the Research and Treatment of Cancer Quality of Life Questionnaire-C30, *OX-CR* controlled-release oxycodone, *OXN-CR* controlled-release oxycodone/naloxone

^a^Patients with available data at week 4. Scores for 22 patients in the OXN-CR group and 28 patients in the OX-CR group at week 4 were missing because these patients discontinued the study

#### Safety

No significant differences were observed between treatment groups with respect to the occurrence rates of any AEs, any ADRs, or any serious ADRs (Table 3). During the study, 55 (85.9%) OXN-CR-treated patients experienced 172 AEs, and 57 (89.1%) OX-CR-treated patients experienced 183 AEs. The most frequently reported AEs were gastrointestinal disorders (e.g., constipation, nausea, anorexia, and vomiting), dizziness, and dyspnea (Table 3).

**Table 3:** Summary of patients in the OXN-CR and OX-CR groups who experienced adverse events (AEs) during the study (safety analysis population)

Variable	OXN-CR [cases (%)]	OX-CR [cases (%)]	*P* value
AEs			
Any AEs	55 (85.9)	57 (89.1)	0.593
Serious AEs	15 (23.4)	28 (43.8)	0.013
Most common AEs^a^			NA
Constipation	30 (46.9)	35 (54.7)	
Nausea	10 (15.6)	18 (28.1)	
Anorexia	8 (12.5)	5 (7.8)	
Vomiting	8 (12.5)	7 (10.9)	
Dizziness	6 (9.4)	14 (21.9)	
Dyspnea	1 (1.6)	7 (10.9)	
ADRs			
Any ADRs	38 (59.4)	41 (64.1)	0.585
Serious ADRs	1 (1.6)	4 (6.3)	0.168
Most common ADRs^a^			NA
Constipation	27 (42.2)	28 (43.8)	
Nausea	7 (10.9)	11 (17.2)	
Dizziness	5 (7.8)	12 (18.8)	

*ADR* adverse drug reaction, *NA* not applicable

^a^AEs or ADRs reported by at least 10% of patients in either treatment group

Most AEs in both groups were grades 1 and 2 in severity [OXN-CR 147 (85.5%) events; OX-CR 156 (85.2%) events]. Ten grade 4 AEs were reported [OXN-CR 4 (2.3%) events; OX-CR 6 (3.3%) events]; however, none was deemed to be related to the study drugs after evaluation by the study physician. Two patients in the OX-CR group experienced grade 5 AEs and died during the study because of cancer progression.

ADRs were reported in 38 (59.4%) patients with 69 events in the OXN-CR group and in 41 (64.1%) patients with 81 events in the OX-CR group. Most ADRs were grades 1 and 2; 2 (2.9%) events in the OXN-CR group and 4 (4.9%) events in the OX-CR group were grade 3. The most frequently reported ADRs were constipation, nausea, and diarrhea (Table 3).

Significantly fewer OXN-CR-treated patients experienced serious AEs than OX-CR-treated patients (23.4% vs. 43.8%, *P* = 0.013) (Table 3). Five patients experienced 8 serious ADRs: 1 patient in the OXN-CR group experienced 1 event of hyperalgesia and 4 patients in the OX-CR group experienced 2 events of limb numbness and 1 event each of anxiety, somnolence/depressed level of consciousness, sweating, tremor, and urinary retention.

Thirteen patients (5 in the OXN-CR group and 8 in the OX-CR group) prematurely discontinued the study because of at least one ADR, specifically dizziness (4 events), nausea (3 events), and vomiting (2 events) in the OXN-CR group, and dizziness (4 events), nausea (4 events), vomiting (3 events), and constipation (3 events) in the OX-CR group. Four other patients (1 in the OXN-CR group and 3 in the OX-CR group) discontinued the study because of an AE that was unrelated to the study medication. No clinically significant abnormalities were observed in clinical laboratory test results, ECGs, or vital signs.

## Discussion

In the present study, we found that OXN-CR shows a similar analgesic efficacy as OX-CR in Korean patients with moderate to severe cancer-related pain. This finding was indicated by the non-inferiority of OXN-CR versus OX-CR in terms of the mean change in NRS pain scores after 4 weeks of treatment. Additionally, the use of analgesic and laxative rescue medications, bowel habit change, QoL, and safety parameters were similar in the OXN-CR and OX-CR groups.

Efficacy and safety results for OXN-CR in the present study are consistent with the findings of a previous phase II study by Ahmedzai et al. [12], in which patients with moderate to severe cancer-related pain (*n* = 185) were randomized to receive either OXN-CR or OX-CR (up to 120 mg/day oxycodone equivalents). Mean scores on the Brief Pain Inventory Short-Form were similar in the OXN-CR and OX-CR groups at baseline (4.16 ± 1.87 and 4.18 ± 1.87) and at week 4 (3.50 ± 1.88 and 3.52 ± 1.80), indicating the non-inferiority of OXN-CR to OX-CR (least squares mean value of difference −0.011; 95% CI −0.47 to 0.45; *P* < 0.010). Additionally, the dose and intake frequency of analgesic rescue medication were generally low in both treatment groups. Overall, the findings from the current study are consistent with those from the phase II study in Caucasian patients [12], providing further evidence of the analgesic efficacy of OXN-CR. Since the average baseline NRS pain scores in both treatment groups were between 4 and 7 (moderate pain), there may be limited generalizability of the findings to populations of patients with more severe pain.

In the current study, most patients reported no change in their bowel habits with either OXN-CR or OX-CR. In the study by Ahmedzai et al. [12], opioid-induced constipation was assessed using the Bowel Function Index (BFI), which is a validated, three-item questionnaire using subjective criteria [19]. The change in the BFI score from baseline to week 4 was significantly better in patients treated with OXN-CR (difference −11.14; 95% CI −19.03 to −3.24; *P* < 0.010). Mean total daily laxative intake (i.e., oral bisacodyl) after 4 weeks was 20% lower in patients treated with OXN-CR compared with those treated with OX-CR, although the difference was not significant (*P* = 0.170) [12].

The BFI was not assessed in the current study, precluding comparison with the earlier phase II study [12]. Assessment of the BFI was not considered because the current study was originally designed to recruit a heterogeneous population of opioid-naïve and opioid-tolerant patients. Furthermore, patient reporting of changes in bowel habits over the previous 4 weeks is a measure that may be subject to recall bias, particularly without a patient diary. In the current study, several reasons could potentially explain the apparent lack of significant improvement in bowel function with OXN-CR versus OX-CR. First, the doses of OXN-CR and OX-CR used here (approximately 30 mg/day oxycodone equivalents) were lower than those used in the phase II study by Ahmedzai et al. (approximately 45 mg/day oxycodone equivalents). The simple Likert scale (“improved,” “unchanged,” or “worsened”) employed in the current study could have been insufficient to detect small or subtle changes in bowel habits, compared with the three-item BFI. Second, interpretation of the results may be complicated by the fact that the current study had a high proportion (40%) of patients with a history of colorectal cancer. By comparison, less than 10% of Caucasian patients in the earlier phase II study had colorectal cancer as the primary cancer [12]. In the present study, bowel habit changes were not found to differ significantly between the treatment groups when only the subset of patients without a history of colorectal cancer was considered. However, it would have been preferable to exclude patients with a history of colorectal cancer from the study altogether because of the expected effects of the disease on bowel function.

Laxative use was originally included as a secondary endpoint in the current study. However, laxative medication was not a study drug and therefore was prescribed as needed, without intent to control the dose used. For better comparability of laxative use between OXN-CR and OX-CR groups, it would have been preferable to stipulate the maximum dose and/or dosing frequency of laxative rescue medication during the study.

At week 4, results for both the general and constipation-specific EORTC QLQ-C30 scores were similar in the OXN-CR and OX-CR groups in the present study. Ahmedzai et al. [12] reported similar results for general QoL in the OXN-CR and OX-CR groups but noted a lower mean constipation-specific QoL subscore (better outcome) in the OXN-CR group compared with the OX-CR group. Patients treated with OXN-CR also reported greater improvement in the score of Patient Assessment of Constipation Symptoms than those treated with OX-CR [12].

In the current study, OXN-CR and OX-CR showed a similar safety profile; the occurrence rate of AEs was not significantly different in the two treatment groups. For both treatments, the most frequently reported AEs were gastrointestinal-related, such as constipation, nausea, and vomiting. These AEs were expected and are listed as common AEs in the product leaflet for oxycodone [20]. Serious ADRs were infrequent and occurred in only 1.6% of OXN-CR-treated patients and in 6.3% of OX-CR-treated patients. No serious ADRs were repeatedly reported in more than one patient in either treatment group.

## Conclusions

We found that, in Korean patients with moderate to severe cancer-related pain, treatment with OXN-CR or OX-CR for 4 weeks showed similar analgesic effects. Further studies in additional populations of Korean patients with cancer-related pain are needed to compare the long-term analgesic effects of OXN-CR and OX-CR treatment, especially in the context of managing constipation symptoms.
